# Protective effects of γ-aminobutyric acid against H_2_O_2_-induced oxidative stress in RIN-m5F pancreatic cells

**DOI:** 10.1186/s12986-018-0299-2

**Published:** 2018-09-03

**Authors:** Xue Tang, Renqiang Yu, Qin Zhou, Shanyu Jiang, Guowei Le

**Affiliations:** 1 State Key Laboratory of Food Science and Technology, Jiangnan University, Wuxi, 214122 Jiangsu China; 2 The Affiliated Wuxi Maternity and Child Health Care Hospital of Nanjing Medical University, Wuxi, 214002 Jiangsu China; 3 School of Food Science and Technology, Jiangnan University, Wuxi, 214122 Jiangsu China

**Keywords:** GABA, Anti-oxidation, Anti-apoptosis, Insulin secretion, RINm5f cells

## Abstract

**Background:**

γ-Aminobutyric acid (GABA) is a major inhibitory neurotransmitter in the central nervous system and reported to maintain the redox homeostasis and insulin secretion function of pancreatic β cells. This study tested the hypothesis that GABA maintains cellular redox status, and modulates glycogen synthase kinase (GSK)-3β and antioxidant-related nuclear factor erythroid 2-related factor 2 (NRF2) nuclear mass ratio in the H_2_O_2_-injured RINm5F cells.

**Methods:**

RINm5F cells were treated with/without GABA (50, 100 and 200 μmol/L) for 48 h and then exposed to 100 μmol/L H_2_O_2_ for 30 min. Viable cells were harvested, and dichloro-dihydro-fluorescein diacetate (DCFH-DA) was used to detect reactive oxygen species (ROS) level; cellular redox status and insulin secretion were measured; cell viability was determined by 3-(4,5-dimethyl-thiazol-2-yl)-2,5-diphenyl tetrazolium bromide (MTT) assay; mitochondrial membrane potential (MMP) was detected by flow cytometry; relative genes levels were analyzed by reverse transcriptase polymerase chain reaction (RT-PCR); western blotting was used to determine protein expression of GSK-3β and p-GSK-3β (Ser9), and nuclear and cytoplasmic NRF2.

**Results:**

H_2_O_2_ increased ROS production, and induced adverse affects in relation to antioxidant defense systems and insulin secretion. These changes were restored by treatment with 100 and 200 μmol/L GABA. In addition, 100 or 200 μmol/L GABA induced membrane depolarization and increased cell viability. These effects were mediated by Caspase-3, Bcl-2 associated X protein (*Bax*) and B-cell lymphoma-2 (*Bcl-2*) expression. Western blotting indicated that GABA inhibited GSK-3β by increasing p-GSK-3β (Ser9) level, and directed the transcription factor NRF2 to the nucleus.

**Conclusion:**

In rat insulin-producing RINm5F cells, GABA exerts its protective effect by regulating GSK-3β and NRF2, which governs redox homeostasis by inhibiting apoptosis and abnormal insulin secretion by exposure to H_2_O_2._

## Background

Type 1 diabetes (T1D) is an autoimmune disease characterized by pancreatic insulin-producing cells, which results in hyperglycemia [1]. At the onset of T1D, > 70% of β cells are destroyed, whereas the residual β-cells most likely represent the only reservoir for the regeneration of islet β-cell mass [2]. Oxidative stress is associated with the development of T1D and represents a central pathophysiological mediator of diabetes [3]. Compared with other tissues of the body, pancreatic β cells have lower levels of antioxidant enzymes, such as superoxide dismutase (SOD), catalase (CAT) and glutathione peroxidase (GSH-PX) activity, hence are more susceptible to pancreatic β-cell reactive oxygen species (ROS) damage [4]. Oxidative stress can also directly damage islet β cells and reduce the sensitivity of peripheral tissues to insulin. This results in an absolute or relative lack of insulin secretion, leading to abnormal blood glucose levels, and therefore is considered a major risk factor in the development and progression of diabetes [5]. Thus, there has been a growing interest in identifying endogenous antioxidant and growth factors for β-cell replication.

γ-Aminobutyric acid (GABA) is synthesized from glutamate by glutamic acid decarboxylase, is a major neurotransmitter in the central nervous system [6]. In the adult brain, GABA induces rapid inhibition in neurons, mainly through the GABAA receptor [7].GABA is also produced by the pancreas, and β cells are the main cells in the pancreas that secrete GABA [8]. GABA and insulin coexist in large dense-core vesicles of human islets, and the release of GABA in β cells is dependent on glucose concentration [9, 10]. GABA activates GABA receptors of pancreatic α cells by a paracrine mechanism, causing cell membrane hyperpolarization, suppression of glucagon, and prevention of high glucose concentration [11, 12]. GABA also activates GABA A receptors by an autocrine mechanism, causing membrane depolarization and increasing insulin secretion [13, 14]. Insulin counter-regulates GABAA receptor function, inhibiting GABA-induced insulin secretion [15], indicating that insulin can dual-directionally regulate islet β-cell GABA secretion.

GABA has a regulatory role in insulin secretion, and many studies have shown that it has a protective effect on islet β cells. Soltani et al. [16] reported that GABA therapy protects NOD mice against T1D. Remarkably, GABA also reverses established diabetes, which is most notable in streptozotocin-induced disease, whereas disease reversal in NOD mice is less prominent. The cellular and molecular mechanisms of these effects may be attributed to induction of membrane depolarization and Ca^2+^ influx by GABA, leading to activation of phosphoinositide 3-kinase (PI3K)/AKT-dependent growth and survival pathways in islet β cells [16]. PI3K/AKT are key molecules in the nuclear factor erythroid 2-related factor 2 (NRF2) -mediated regulation of antioxidative proteins. PI3K/AKT/NRF2 control the basal and induced expression of an array of antioxidant response-element-dependent genes to regulate the physiological and pathophysiological outcomes of oxidant exposure [17] suggesting that GABA improves antioxidative capacity under oxidative stress conditions. However, the role of GABA signaling in the regulation of β-cell antioxidant capability remains largely unknown. GABA has antioxidative activity, therefore, we hypothesized that GABA treatment can exert protection against H_2_O_2_-induced oxidative injury in islet β cells.

## Material and methods

### RINm5F culture: H_2_O_2_ exposure and GABA treatment

Mouse insulinoma RINm5F cells were cultured in RPMI 1640 medium (ICN, Eschwege, Germany) containing fetal bovine serum (10% *v*/v). All cell culture media were supplemented with 2 mmol/L L-glutamine, 100 mg/mL penicillin and 50 mg/mL streptomycin. Cells were maintained at 37 °C under humidified conditions of 95% air and 5% CO_2_.

On reaching 80% confluence, cells were treated (or not) with GABA (50, 100 or 200 μmol/L) alone for 48 h. After every 24 h, fresh aliquots of GABA were added to culture medium in all the experiments. Then, the cells were exposed to H_2_O_2_ (100 μmol/L) for 30 min in the presence or absence of GABA. Control cultures were grown under the same culture condition as treated cells, but in the absence of GABA and H_2_O_2._

### Analysis of the redox balance

For measurement of ROS production, control and treated cells were cultured in 96-well plates at a density of 1 × 10^4^ cells per well, and incubated in 100 μL medium with 1‰ dichloro-dihydro-fluorescein diacetate (DCFH-DA) at 37 °C for 30 min. Mean fluorescence intensity (excitation wavelength 525 nm, emission wavelength 488 nm) was read by microtiter plate reader (Molecular Devices, Sunnyvale, CA, USA). Data were expressed as the mean percentage of viable cells versus the controls.

Cell lysates isolated from RINm5F cells were tested for total antioxidant capacity (T-AOC), SOD, CAT and GSH-PX activity, and malondialdehyde (MDA) content with the appropriate test kits obtained from Nanjing Jiancheng Bioengineering Institute (Nanjing, China).

### Insulin secretion

After incubation with H_2_O_2_, in the presence or absence of GABA, the insulin secretion was assessed as previously described [18]. Cells were kept in 5.5 mmol/L glucose until the day of the experiment, then challenged with 22 mmol/L glucose or re-exposed to 5.5 mmol/L glucose for 1 h. The level of insulin in the culture medium was measured by mouse insulin ELISA kit (Nanjing Jiancheng Bioengineering Institute, Nanjing, China) and normalized for the total cellular protein content detected in the pellet of each individual culture according to the Bradford method (Bio-Rad Laboratories, Stockholm, Sweden).

### Mitochondrial membrane potential (MMP) measurements

The MMP of control and treated cells was determined by staining RINm5F cells with JC-1 (5,5′,6,6′-tetrachloro-1,1′,3,3′-tetraethyl-benzimidazolylcarbocyanine chloride), a lipophilic, cationic dye that exhibits a fluorescence emission shift upon aggregation from 530 nm (green monomer) to 590 nm (red J aggregates) [19]. In healthy cells with high MMP, JC-1 entered the mitochondrial matrix in a potential-dependent manner and formed aggregates. Cells were collected and stained by JC-1. After staining, cells were rinsed in 3× phosphate-buffered saline. The MMP was measured by flow cytometry (BD Biosciences, San Jose, CA, USA).

### MTT assays

Cell viability was measured using blue formazan that was metabolized from colorless MTT (3-(4,5-dimethyl-thiazol-2-yl)-2,5-diphenyl tetrazolium bromide) by mitochondrial dehydrogenases, which are active only in live cells. Cells were plated in 96-well plates at 70% confluence. After 12 h, cells were exposed to GABA and H_2_O_2_. MTT solution was added to the culture medium, and after 4 h, 150 μL of solubilization solution was added to each well and absorption values read at 490 nm on a microtiter plate reader (Molecular Devices). Data were expressed as the mean percentage of viable cells versus the controls.

### RNA extraction and reverse transcriptase polymerase chain reaction (RT-PCR)

Total RNA was extracted from the cells with TRIzol reagent (Invitrogen, Carlsbad, CA, USA). Total RNA was reverse-transcribed into cDNA using oligo-dT as a primer and MMLV reverse transcriptase, and 2 μL of the cDNA template was used to amplify the different mRNAs. ABI PRISM 7500 Sequence Detection System was used to perform real-time PCR. The PCR conditions were 95 °C for 5 min, followed by 45 cycles of 95 °C for 20 s, 62 °C for 30 s, and 72 °C for 20 s. The sequences of the oligonucleotide primers used in this study were as follows: *Caspase-3* (forward, CTTATCCTTATACAAATCAGCTCGG; reverse, TCAAACCACATTCTCTCCAACTACA); Bcl-2 associated X protein (*Bax*) (forward, TGGAGATGAACTGGACAGCAATAT; reverse, GCAAAGTAGAAGAGGGCAACCAC); B-cell lymphoma-2 (*Bcl-2*) (forward, AACTCTAACTGTGCTTTGAAGGTGA; reverse, AGCTCAGAAGAGAACTTTAGTGGCT); Pancreatic and duodenal homeobox 1 (*Pdx-1*) (forward, CTCACCTCCACCACCACCTTCC; reverse, CACCTCCTGCCCACTGGCCTTT), v-maf musculoaponeurotic fibrosarcoma oncogene homolog A (*Mafa*) (forward, CATCACCACCACGGAGGCT; reverse, CGCACGGACATGGATACCA); Gamma-aminobutyric acid receptor subunit alpha2 (*Gabra2*) (forward, GCTTGGGACGGGAAGAGTGTAGT; reverse, GGAAAGATTCGGGGCATAGTTGG), β-actin (forward, GGGTCAGAAGGACTCCTATG; reverse, GTAACAATGCCATGTTCAAT). The relative expression levels of the target genes were calculated as a ratio to the housekeeping gene β-actin. Melting curve analysis was performed to assess the specificity of the amplified PCR products.

### Western blotting

The whole cell extracts were obtained by using cell lysis buffer (Cell Signaling Technology, Beverly, MA, USA) with 0.5% protease inhibitor cocktail (Sigma, St Louis, MO, USA) and 1% phosphatase inhibitor cocktail I (Sigma). Nuclear and cytosolic fractions were separated using a nuclear and cytoplasmic protein extraction kit (Beyotime Biotechnology, Shanghai, China). Protein levels were measured using a bicinchoninic acid protein determination kit (Keygen Biotech, Nanjing, China). Total protein (60 μg) was applied to a 12% SDS-polyacrylamide gel. After electrophoresis and transfer to polyvinylidene fluoride membranes, the membranes were washed in Tris-buffered saline containing 0.1% Tween 20, and incubated with a primary antibody (rabbit anti-glycogen synthase kinase (GSK)-3β, rabbit anti-p-GSK-3β (Ser 9), rabbit or rabbit anti-NRF2 diluted at 1:1000; Santa Cruz Biotechnology, Santa Cruz, CA, USA). Membranes were incubated with a secondary antibody (1:1000), and immunostained bands were detected with a ProtoBlot II AP System and a stabilized substrate (Promega, Madison, WI, USA). GADPH and histone H3 was used as an internal control.

### Statistical analysis

Data are representative of mean ± SD with three repeats. Comparisons across groups were carried out using one-way analysis of variance with the post-hoc Duncan’s test. *p* < 0.05 was considered to be statistically significant. Analysis of the data was achieved using SPSS version 13 (SPSS, Chicago, IL, USA).

## Results

### GABA reduces ROS level and restores oxidative redox status

**Fig. 1:**
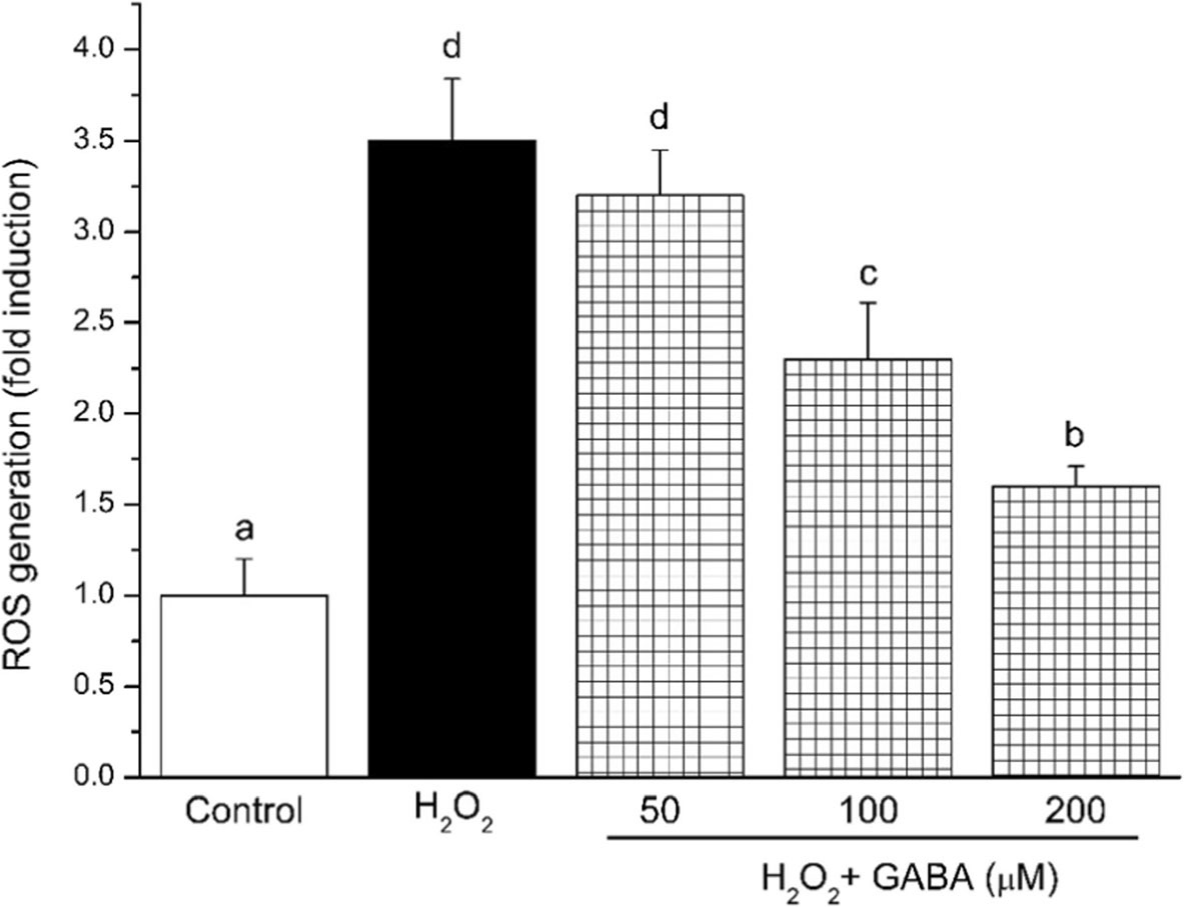
GABA inhibits H_2_O_2_-induced ROS production. Results are the mean of at least three separate ROS assay. Values are means (*n* = 12), with standard errors represented by vertical bars. Means with different lower-case letters are significantly different (*P* < 0.05). Control, control medium; H_2_O_2_, medium containing 100 μM H_2_O_2_; H_2_O_2_ + (50 μM, 100 μM, 200 μM) GABA, medium containing 100 μM H_2_O_2_ and GABA; ROS, reactive oxygen species

To evaluate the protective activity of GABA against H_2_O_2_-induced oxidative stress, we analyzed ROS production by DCFH-DA assay. Exposure to 100 μmol/L H_2_O_2_ resulted in significant overproduction of ROS, with respect to cells cultured in the absence of H_2_O_2_ (Fig. 1). Importantly, 100 and 200 μmol/L GABA prevented H_2_O_2_-induced oxidative stress by significantly decreasing ROS production (*P* < 0.05).

We next investigated the cellular antioxidant capacity (Table 1). H_2_O_2_ significantly decreased the activity of CAT, SOD, GSH-PX and T-AOC but increased MDA content in RINm5f cells (*P* < 0.05). When cells were treated with 100 or 200 μmol/L GABA, T-AOC, and CAT, SOD and GSH-PX activity were significantly increased and MDA content was significantly reduced (*P* < 0.05) compared to the H_2_O_2_ group. These changes were particularly seen in the H_2_O_2_ + 200 μmol/L GABA group.

**Table 1:** GABA causes an increment in the antioxidant status of RINm5F exposed to H_2_O_2_^*^

	T-AOC	CAT	SOD	GSH-Px	MDA
	(U/mg prot)	(U/mg prot)	(U/mg prot)	(U/mg prot)	(nM/mg prot)
Control	1.20 ± 0.15^d^	8.65 ± 0.63^d^	16.31 ± 1.21^d^	138.91 ± 11.02^d^	3.18 ± 0.26^e^
H_2_O_2_	0.26 ± 0.06^a^	2.30 ± 0.19^a^	4.49 ± 0.45^a^	57.31 ± 5.97^a^	6.50 ± 0.52^a^
H_2_O_2_ + 50 μmol/L GABA	0.40 ± 0.05^a^	2.57 ± 0.45^ab^	5.57 ± 0.48^a^	63.39 ± 4.02^a^	6.17 ± 0.54^ab^
H_2_O_2_ + 100 μmol/L GABA	0.59 ± 0.07^b^	3.19 ± 0.22^b^	8.29 ± 0.57^b^	75.80 ± 8.31^b^	5.52 ± 0.29^bc^
H_2_O_2_ + 200 μmol/L GABA	0.76 ± 0.07^c^	4.71 ± 0.38^c^	11.71 ± 0.74^c^	91.00 ± 10.07^c^	5.02 ± 0.29^cd^

Control, control medium; H_2_O_2_, medium containing 100 μmol/L H_2_O_2_; H_2_O_2_ + (50 μmol/L, 100 μmol/L, 200 μmol/L) GABA, medium containing 100 μmol/L H_2_O_2_ and GABA; *T-AOC* Total antioxidant capacity, *CAT* Catalase, *SOD*, Superoxide dismutase, *GSH-PX* Glutathione peroxidase, *MDA* Malondialdehyde

*Values are expressed as mean ± SD (*n* = 8) of three separate experiments. Means with different superscript letters within a same column gare significantly different (*P* < 0.05)

### GABA inhibits mitochondrial damage and cell death

**Fig. 2:**
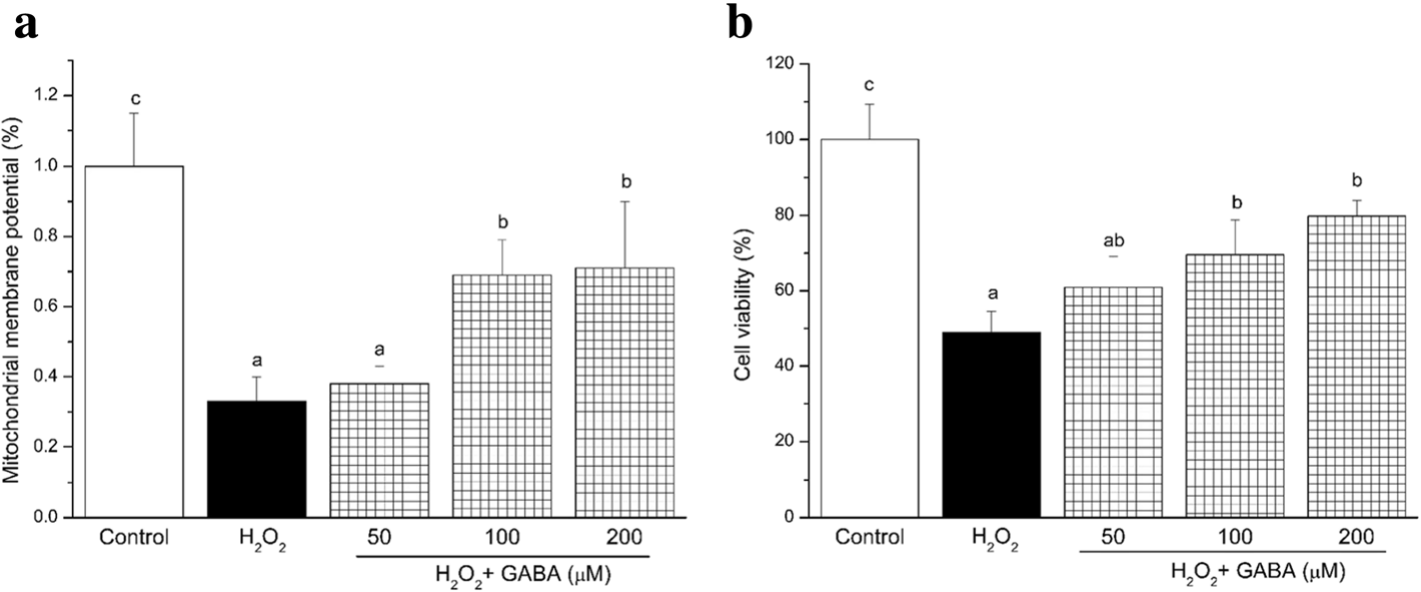
GABA inhibits mitochondrial damage and cell death. **a** mitochondrial membrane potential (MMP). Biparametric flow cytometry analysis after staining of living cells with JC-1. **b** Cell viabilities were analyzed by MTT assay. Data are shown as the mean ± SD of three independent experiments (*n* = 4). Means with different lower-case letters are significantly different (*P* < 0.05). Control, control medium; H_2_O_2_, medium containing 100 μM H_2_O_2_; H_2_O_2_ + (50 μM, 100 μM, 200 μM) GABA, medium containing 100 μM H_2_O_2_ and GABA

We used flow cytometry to measure MMP by staining RINm5f cells with JC-1 (Fig. 2a). MTT assays were also used to measure cell viability (Fig. 2b). The cells treated with 100 μmol/L H_2_O_2_ showed significantly lower MMP compared to the control group (*P* < 0.05). A similar tendency was observed for cell viability. Pre-treatment with 50, 100 and 200 μmol/L GABA partially restored MMP and cell viability but they remained lower than in the control group (*P* < 0.05). Administration of GABA prevented the effect of H_2_O_2_ on this depolarization of MMP and cell death.

**Fig. 3:**
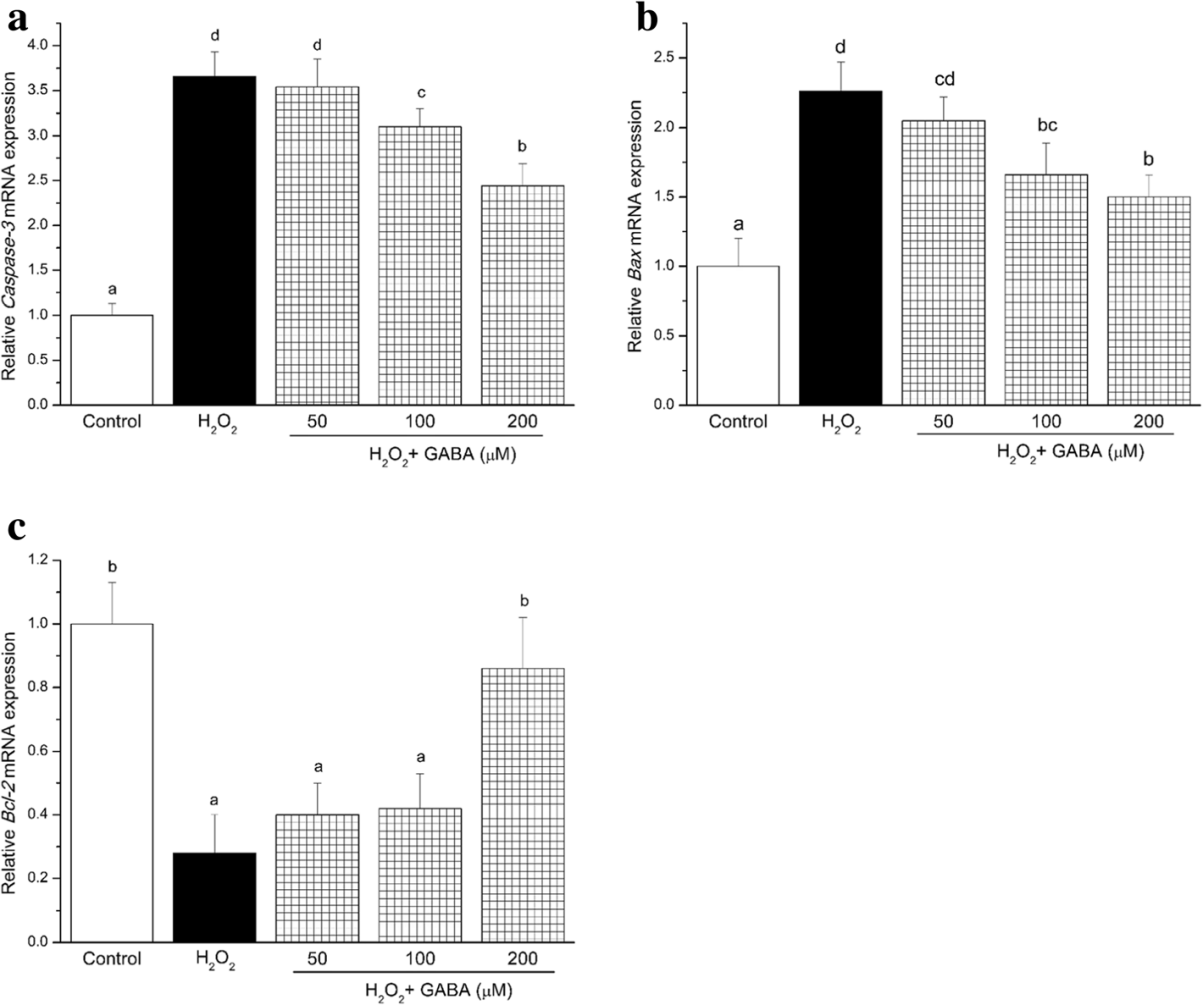
GABA regulates the mRNA expression of the major pro- and anti-apoptotic factors in RINm5F. Real-time quantitative measurement of Caspase-3 **a** Bax **b** and Bcl-2 **c** mRNA levels in RINm5F cells. Data are shown as the mean ± SD of three independent experiments (n = 4). Means with different lower-case letters are significantly different (*P* < 0.05). Control, control medium; H_2_O_2_, medium containing 100 μM H_2_O_2_; H_2_O_2_ + (50 μM, 100 μM, 200 μM) GABA, medium containing 100 μM H_2_O_2_ and GABA

RT-PCR was used to determine *Caspase-3*, *Bax* and *Bcl-2* gene mRNA expression in RINm5f cells (Fig. 3). We found that 100 μmol/L H_2_O_2_ induced a significant increase in *Caspase-3* and *Bax* expression, and a decrease in *Bcl-2* expression. However, 100 or 200 μmol/L GABA prevented the increase in H_2_O_2_-induced *Caspase-3* and *Bax* expression, and restored *Bcl-2* expression (*P* < 0.05). GABA inhibited cell death by regulating the molecular targets involved in the apoptotic cascade inactivated by different apoptotic stimuli.

### GABA protection of insulin-secreting RINm5F cells is associated with an increase of Pdx-1, MaFA and Cabra2 expression

To assess functional modifications, glucose-dependent secretion of insulin was evaluated in RINm5F cells in the presence or absence of GABA (Table 2). Insulin release from cells exposed only to H_2_O_2_ was markedly impaired in the absence of GABA (*P* < 0.05). Adding GABA did not modify basal insulin release (at 5.5 mmol/L glucose) as compared with GABA-untreated cells. When challenged with 22 mmol/L glucose, cells treated with 100 or 200 μmol/L GABA+H_2_O_2_ showed a significant increase in insulin release, as compared with H_2_O_2_-treated cells (*P* < 0.05).

**Table 2:** Insulin secretion from RINm5F cells in response to glucose (5.5 and 22 mmol/L) concentration^*^

	Glucose	
	5.5 mmol/L	22 mmol/L
Control	25.45 ± 2.01	58.98 ± 7.27^d^
H_2_O_2_	20.14 ± 2.50	19.46 ± 3.17^a^
H_2_O_2_ + 50 μmol/L GABA	26.62 ± 1.65	22.32 ± 4.41^a^
H_2_O_2_ + 100 μmol/L GABA	24.71 ± 2.94	31.88 ± 4.30^b^
H_2_O_2_ + 200 μmol/L GABA	25.92 ± 3.16	38.70 ± 3.97^c^

Control, control medium; H_2_O_2_, medium containing 100 μmol/L H_2_O_2_; H_2_O_2_ + (50 μmol/L, 100 μmol/L, 200 μmol/L) GABA, medium containing 100 μmol/L H_2_O_2_ and GABA

^*^Values are expressed as mean ± SD (n = 8) of three separate experiments. Means with different superscript letters within a same column gare significantly different (*P* < 0.05)

**Fig. 4:**
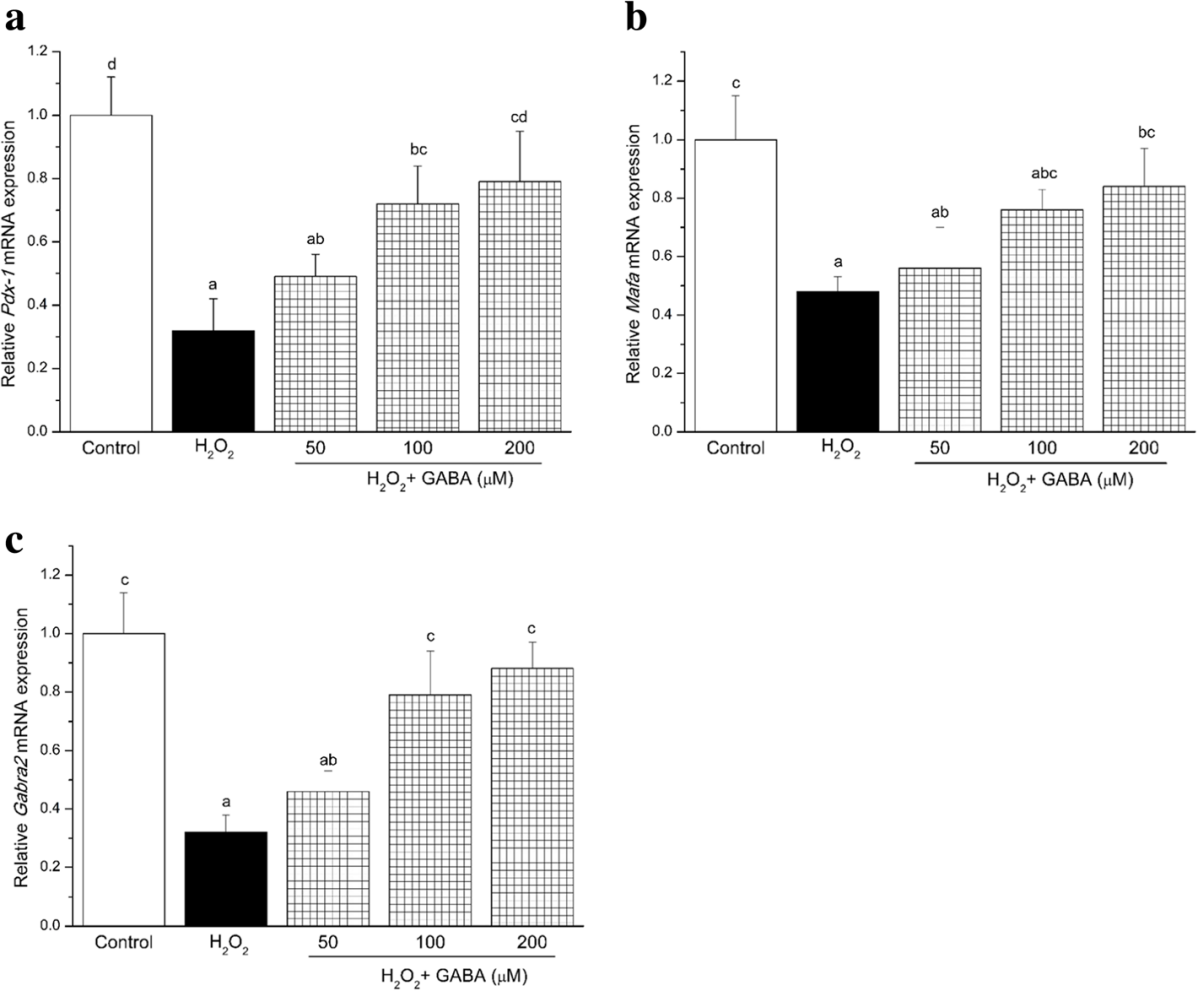
GABA modulates Pdx-1 **a** Mafa **b** and Gabra2 **c** mRNA expression. Results are the mean of at least three separate experiments. Values are means (n = 4), with standard errors represented by vertical bars. Means with different lower-case letters are significantly different (*P* < 0.05). Control, control medium; H_2_O_2_, medium containing 100 μM H_2_O_2_; H_2_O_2_ + (50 μM, 100 μM, 200 μM) GABA, medium containing 100 μM H_2_O_2_ and GABA

RT-PCR analysis of RINm5F cells revealed that GABA induced upregulation of transcription activating factors *Mafa* and *Pdx-1*, which are involved in regulation of glucose-stimulated insulin secretion (Fig. 4a and b). GABA A receptor (*Gabra2*) was also upregulated by 100 and 200 μmol/L GABA when the cells were exposed to H_2_O_2_ (Fig. 4c). These data suggest that the GABA protects insulinoma cells from H_2_O_2_ by regulating the different molecular targets involved in glucose-stimulated insulin secretion.

### GABA exerts antioxidative effects through GSK/3β and NRF2

**Fig. 5:**
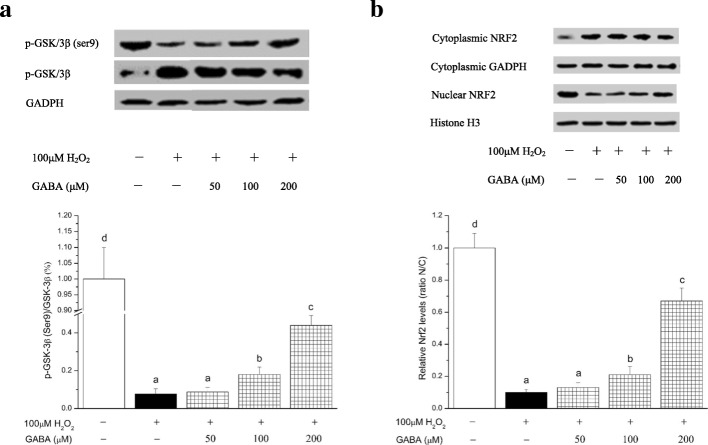
GABA regulates GSK-3β and prevents the H_2_O_2_-induced redistribution of NRF2 towards the nucleus. GSK-3β and p-GSK/3β (ser9) protein **a** and cytosolic and nuclear NRF2 protein **b** were evaluated by western blot. The relative amount of NRF2 was calculated as the ratio between nuclear and cytosolic levels (ratio N/C). Data are shown as the mean ± SD of three independent experiments (n = 4). Means with different lower-case letters are significantly different (*P* < 0.05)

GSK-3β is tightly regulated by the survival pathway represented by PI3K/AKT. Activation of PI3K/AKT leads to inactivation of GSK/3β by phosphorylation of its Ser9 residue [20]. Phosphorylation of GSK/3β at Ser9 is associated with regulation of many important metabolic and signaling proteins, structural proteins and transcription factors [21]. To determine whether GABA-regulated cell antioxidative activity was associated with phosphorylation of GSK/3β, western blotting with antibody specific for p-GSK-3β (Ser9) was performed (Fig. 5a). GABA (100 and 200 μmol/L) downregulated the protein level of GSK-3β, while phosphorylation at Ser9 was upregulated by GABA, for the indicated concentrations. GSK-3β is the main protein responsible for maintaining NRF2 in the cytoplasm. Inhibition of NRF2 by GSK-3β limits the cell response to oxidative stress [22]. As shown in Fig. 5b, H_2_O_2_ induced low accumulation of NRF2 in the nucleus. GABA treatment of RINm5F cells at a concentration of 100 and 200 μmol/L gradually increased nuclear NRF2. A maximum nuclear mass ratio was achieved with 200 μmol/L GABA. The results indicate that GABA prevents, at least partially, the H_2_O_2_-induced cytosolic localization of NRF2.

## Discussion

GABA is the primary inhibitory neurotransmitter in the mammalian central nervous system, and activation of GABAA receptors (GABAARs) by GABA tends to decrease neuronal excitability [23]. GABA is mainly synthesized from glutamic acid by glutamic acid decarboxylase (GAD) [24]. The GABA signalling system is an integral part of a system in human islets maintaining glucose homeostasis [25].

Human islets express GABA A and B receptors [26, 27]. On the one hand, GABA suppress glucagon secretion in islet cells by activating GABAA receptors [27]. On the other hand, GABA can also be activated by autocrine GABA A receptors, causing cell membrane depolarization and increasing insulin secretion [28]. Robertson et al. [29] have reported that ROS damage the nuclei and mitochondria of islet β cells, and reduce *Pdx-1* gene expression and its binding activity to DNA by inhibiting activity of the insulin gene promoter, which further reduces insulin gene expression, leading to decreased insulin synthesis. In the present study, oxidative stress was generated by the addition of H_2_O_2_ as a direct oxidant. Insulin release from cells exposed only to H_2_O_2_ was markedly decreased as compared to the control group. GABA induces mRNA expression of GABA A2 receptor and transcription activating factors *Mafa* and *Pdx-1*, which is involved in the regulation of glucose-stimulated insulin secretion. GABA also improves insulin secretion and viability of RIN5mF cells. When GABA is given to mice with T1D, it restores β cells, completely reverses the symptoms of hyperglycemia and cures diabetes, associated with elevated insulin levels and reduced glucagon levels [16]. These effects of GABA could be attributed to its synergy of insulin by activating the PI3K/AKT pathway and increasing β-cell proliferation and survival [15].

Oxidative stress is considered an important mediator of cellular damage following prolonged exposure of pancreatic β cells to elevated levels of H_2_O_2_ [30]. Our observations, consistent with previous reports [28, 29], demonstrated that exposure to H_2_O_2_ resulted in significant overproduction of ROS in RINm5F cells when compared with untreated cells. Our results also showed that H_2_O_2_ strongly reduced T-AOC and activity of CAT, SOD and GSH-PX antioxidant enzymes in RIN5mF cells, while it increased the level of MDA, a lipid oxidation product. It has been demonstrated that pancreatic β cells are especially sensitive to oxidative stress because of their low levels of antioxidant enzymes [31]. Several studies have shown that β-cell death induced by oxidative stress may play an important role in pathogenesis of both type 1 and type 2 diabetes [32, 33]. Antioxidant treatment may improve impaired β-cell function in response to high glucose concentration [4].

Our data suggest that GABA exerts its protective effect by strengthening the intrinsic antioxidant defenses of RINm5F cells through modulation of antioxidant enzymes, thus protecting them by acting on the redox status. This hypothesis is supported by previous studies showing that in red algae amino acid induced by nerve injury research, GABA has a protective role by reducing cellular ROS and MDA levels in kainic acid-induced status epilepticus [34]. GABA inhibits neural NO synthesis in rat ileum by GABA A and GABA C (Aρ) receptor-mediated mechanisms [35]. GABA significantly increases SOD and GSH-PX activity, reduces MDA level, and scavenges free radicals in vivo and in vitro [36].

ROS-induced oxidative stress is an important cause of apoptosis. Long-term high blood sugar and oxidative damage reduce islet β-cell synthesis and secretion of insulin, and reduce the number of islet β cells; both of which increase the development of diabetes [5]. It has been demonstrated that loss of MMP is indicative of apoptosis and precedes phosphatidylserine externalization and caspase activation [37]. In the present study, the possibility that the effect of GABA might involve apoptotic pathways was suggested by measurements of MMP. Our data showed that GABA strongly attenuated depolarization of the mitochondrial membrane in a significant number of H_2_O_2_-treated cells. Evidence for the possibility that GABA reduces apoptosis was obtained by data showing a decrease in *Caspase-3* expression in response to H_2_O_2_. Caspase-3 has been identified as a key mediator of apoptosis of mammalian cells. It can activate death protease and catalyze the specific cleavage of many key cellular proteins [38]. Inhibition of Caspase-3 with GABA abolished the increase in cell death induced by H_2_O_2_, which suggests that part of the GABA effect is associated with regulation of *Caspase-3* expression. In human and rodent islets, members of the Bcl-2 family modulate apoptosis, with the Bax/Bcl-2 ratio determining cell susceptibility to apoptosis [39]. It has been suggested that overexpression of Bcl-2 protein enhances islet viability [40]. The present study showed that 50 or 200 μmol/L GABA significantly upregulated *Bcl-2* expression in RIN5mF cells. Conversely, *Bax* expression was significantly decreased in cells treated with GABA, as compared to the H_2_O_2_ group. Moreover, Bcl-2 protected cells from H_2_O_2_-induced oxidative death through regulation of cellular antioxidant enzymes (e.g., SOD and CAT) [41, 42]. These results indicate that GABA-induced antioxidative activity in pancreatic β cells may involve mechanisms dependent upon Bcl-2. This requires further investigation.

GSK-3β is involved in regulation of glycogen metabolism. It not only affects glycogen synthesis, but also gene transcription, cell division and multiplication, and plays an indispensable role in the process, which involves in many diseases occurrence and development [43, 44]. GSK-3β overexpression inhibits β-cell proliferation in mice, and induces diabetes [45]. On the contrary, inhibition of GSK-3β prevents the onset of diabetes by improving glucose tolerance and β-cell function [46]. Regulation of GSK-3β activity is critically dependent on the phosphorylation state of its Ser9 residue, which is located at the pseudosubstrate domain. Phosphorylation of Ser9 by several kinases, including AKT, results in inhibition of GSK-3β activity [47]. We showed that p-GSK-3β (Ser9) level was increased by GABA when the cells were exposed to H_2_O_2_, suggesting that GABA inhibits GSK-3β activity to improve β-cell function.

Oxidative stress has been shown to regulate PI3K/AKT and, consequently, to alter the downstream signaling events in cultured cells [48]. The PI3K/AKT/GSK-3β axis is essential for the H_2_O_2_-induced nuclear translocation of NRF2. H_2_O_2_ downregulates AKT and activates GSK-3β, together with relocation of NRF2 back to the cytosol [22]. GSK-3β is the main protein responsible for maintaining NRF2 in the cytoplasm [43]. In the present study, we explored the possibility that GABA might regulate the nuclear–cytoplasmic shuttling cycle of NRF2. H_2_O_2_ treatment increased the amount of cytosolic NRF2. However, when GABA was added, NRF2 was redistributed mostly to the nucleus, suggesting that GABA produces high accumulation of NRF2 in the nucleus, thus restoring oxidative redox status under oxidative stress and maintaining cellular function.

## Conclusions

In conclusion, Our results show that GABA inactivates GSK-3β, with subsequent redistribution of NRF2 towards the nucleus. This may represent a mechanism underlying its in vitro effects in promoting β-cell antioxidant capacity, survival and function.

## Abbreviations

GABA: γ-Aminobutyric acid
CAT: Catalase
GSH-PX: Glutathione peroxidase
GSK-3β: glycogen synthase kinase-3β
MDA: Malondialdehyde
NRF2: Nuclear factor erythroid 2-related factor 2
ROS: Reactive oxygen species
SOD: Superoxide dismutase
T1D: Type 1 diabetes
T-AOC: Total antioxidant capacity
